# Demographic and Clinical Profile of Patients with Osteogenesis Imperfecta Hospitalized Due to Coronavirus Disease (COVID)-19: A Case Series of 13 Patients from Brazil

**DOI:** 10.3390/healthcare13151779

**Published:** 2025-07-23

**Authors:** Luana Lury Morikawa, Luiz Felipe Azevedo Marques, Adriele Evelyn Ferreira Silva, Patrícia Teixeira Costa, Lucas Silva Mello, Andrea de Melo Alexandre Fraga, Fernando Augusto Lima Marson

**Affiliations:** 1 Faculty of Medicine, Nove de Julho University (UNINOVE, Which Stands for the Portuguese Universidade Nove de Julho), Guarulhos 07013-110, SP, Brazil; luanamorikawa@uni9.edu.br; 2 Laboratory of Molecular Biology and Genetics, Postgraduate Program of Health Sciences, São Francisco University (USF, Which Stands for the Portuguese Universidade São Francisco), Bragança Paulista 12916-900, SP, Brazil; luiz.azevedo@mail.usf.edu.br (L.F.A.M.); adrieleferreirasilva@gmail.com (A.E.F.S.); patricia.costa@usf.edu.br (P.T.C.); lucas.silva.mello@mail.usf.edu.br (L.S.M.); 3 Laboratory of Clinical and Molecular Microbiology, Postgraduate Program of Health Sciences, São Francisco University (USF, Which Stands for the Universidade São Francisco), Bragança Paulista 12916-900, SP, Brazil; 4 LunGuardian Research Group—Epidemiology of Respiratory and Infectious Diseases, Postgraduate Program of Health Sciences, São Francisco University (USF, Which Stands for the Universidade São Francisco), Bragança Paulista 12916-900, SP, Brazil; andreafrag@gmail.com; 5 Department of Pediatrics, School of Medical Sciences, University of Campinas (UNICAMP, Which Stands for the Portuguese Universidade de Campinas), Campinas 13083-894, SP, Brazil

**Keywords:** epidemiology, medical genetics, osteogenesis imperfecta, pandemic, SARS-CoV-2

## Abstract

**Background:** Osteogenesis imperfecta (OI) is a rare genetic connective tissue disorder characterized by bone fragility, most often caused by pathogenic variants in type I collagen genes. In this context, we aimed to describe the clinical and epidemiological characteristics of patients with OI who were hospitalized for coronavirus disease (COVID)-19 in Brazil between 2020 and 2024. **Methods:** We conducted a retrospective descriptive analysis using data from the Brazilian Unified Health System (SUS, which stands for the Portuguese *Sistema Único de Saúde*) through the Open-Data-SUS platform. Patients with a confirmed diagnosis of OI and hospitalization due to COVID-19 were included. Descriptive statistical analysis was performed to evaluate demographic, clinical, and outcome-related variables. We included all hospitalized COVID-19 cases with a confirmed diagnosis of OI between 2020 and 2024. **Results:** Thirteen hospitalized patients with OI and COVID-19 were identified. Most were adults (9; 69.2%), male (7; 53.8%), self-identified as White (9; 69.2%), and all were residents of urban areas (13; 100.0%). The most frequent symptoms were fever (10; 76.9%), cough (9; 69.2%), oxygen desaturation (9; 69.2%), dyspnea (8; 61.5%), and respiratory distress (7; 53.8%). Two patients had heart disease, one had chronic lung disease, and one was obese. As for vaccination status, five patients (38.5%) had been vaccinated against severe acute respiratory syndrome coronavirus 2 (SARS-CoV-2). Four patients (30.8%) required admission to an intensive care unit (ICU), and six (46.2%) required noninvasive ventilatory support. Among those admitted to the ICU, only two required invasive mechanical ventilation. The clinical outcome was death in two cases (15.4%). Both patients were male, White, and had not been vaccinated against SARS-CoV-2. One was 47 years old, was not admitted to the ICU, but required noninvasive ventilation. Despite the underlying condition most patients had favorable outcomes, consistent with an international report. **Conclusions:** This is the first report to describe the clinical and epidemiological profile of patients with OI hospitalized for COVID-19 in Brazil, providing initial insights into how a rare bone disorder intersects with an acute respiratory infection. The generally favorable outcomes observed—despite the underlying skeletal fragility—suggest that individuals with OI are not necessarily at disproportionate risk of severe COVID-19, particularly when appropriately monitored. The occurrence of deaths only among unvaccinated patients underscores the critical role of SARS-CoV-2 vaccination in this population. Although pharmacological treatment data were unavailable, the potential protective effects of bisphosphonates and vitamin D merit further exploration. These findings support the need for early preventive strategies, systematic vaccination efforts, and dedicated clinical protocols for rare disease populations during infectious disease outbreaks.

## 1. Introduction

Osteogenesis imperfecta (OI) is a rare genetic connective tissue disorder, primarily caused by abnormalities in the synthesis or processing of type I collagen [1]. OI is classified into five main types (I to V), based on the pathogenic variants involved and the pattern of genetic inheritance [2]. Type I, known as non-deforming OI, is the most common, typically inherited in an autosomal dominant pattern and generally results from pathogenic variants in the *COL1A1* (Collagen Type I Alpha 1 Chain) and *COL1A2* (Collagen Type I Alpha 2 Chain) genes, although it may also be linked to X-linked inheritance involving the *PLS3* (plastin-3) gene. The other types include: Type II (perinatal lethal form), Type III (progressive deforming form), Type IV (moderate form), and Type V (associated with calcification of interosseous membranes or hypertrophic callus formation) [1,2]. Currently, a good correlation between genotypic and phenotypic findings has been described in patients with OI, supporting the use of genetic panels to optimize disease diagnosis [3,4].

Also known as brittle bone disease, OI is a rare, clinically and genetically heterogeneous systemic disorder characterized by increased bone fragility and a high lifetime risk of fractures, primarily due to defects in the production or structure of type I collagen [5,6]. Affected individuals often present with low bone mass and a range of phenotypic features including blue sclerae, joint hypermobility, dentinogenesis imperfecta, skeletal deformities, and, in more severe cases, cardiovascular and pulmonary complications [5,6,7,8]. Diagnosis is typically clinical, based on history, physical examination, and radiographic abnormalities findings, as no single confirmatory test or universally accepted clinicopathological classification exists [8]. The estimated global incidence of OI is approximately 1 in 15,000 to 20,000 live births [1,9]. Despite international advances in understanding the disease, epidemiological data on OI in Brazil remain scarce and mainly focus on genetic analysis [10,11,12], and to our current knowledge, no studies have specifically described the clinical and epidemiological characteristics of Brazilian patients with OI affected by emerging infectious diseases such as coronavirus disease (COVID)-19. This gap in the literature limits the development of evidence-based public health policies and clinical management strategies tailored to this vulnerable population.

The COVID-19 pandemic caused by the severe acute respiratory syndrome coronavirus 2 (SARS-CoV-2) rapidly affected populations worldwide, including in Brazil [13]. Although clinical presentations vary from asymptomatic to severe cases involving respiratory failure, multiorgan dysfunction, and death [14], the severity of illness is known to increase in individuals with underlying endocrine, metabolic, and immunological disorders [15,16]. Recent studies have also identified a distinct osteometabolic phenotype in COVID-19, marked by hypocalcemia, chronic vitamin D deficiency, impaired parathyroid hormone response, and increased bone fragility, which predispose individuals to skeletal complications such as vertebral fractures and are associated with worse clinical outcomes [14,17].

Moreover, SARS-CoV-2 infects target cells through the binding of the spike (S) protein to the carboxypeptidase receptors of the angiotensin-converting enzyme (ACE2), leading to its downregulation and consequently to increased levels of angiotensin II [18,19]. This increase promotes a pro-inflammatory state—intensified by the cytokine storm—and exerts a direct effect on bone metabolism. Angiotensin II has osteoclastogenic potential by inducing the expression of Receptor Activator of Nuclear Factor Kappa-B Ligand (RANKL) in osteoblasts, favoring the differentiation of pre-osteoclasts into mature osteoclasts and thus promoting increased bone resorption.

Different mechanisms of action have been hypothesized as responsible for the presence of hypocalcemia in patients affected by COVID-19 [20], such as calcium-dependent viral mechanisms determining intra- and extracellular cation dysmetabolism, calcium deposits in tissues, acute calcium precipitation in muscles, role of calcium in coagulation and prothrombotic state, acute malnutrition during critical illness, high levels of unsaturated and unbound fatty acids in inflammatory responses, and high prevalence of hypovitaminosis D with impaired compensatory response of parathyroid hormone. The increase in circulating cytokines together with a systemic inflammatory response in the body caused by SARS-CoV-2 can lead to a deficiency of the parathyroid gland, which can also be directly affected by the virus, since it is reported that it can bind to its ACE2 receptors. Vitamin D deficiency has been associated as a possible contributor to the endocrine phenotype of COVID-19, also associated with comorbidities in common with the disease, such as older age, male gender, diabetes mellitus, visceral obesity, and hypocalcemia. Patients hospitalized for COVID-19 had hypovitaminosis D, which is associated with the risk of fractures and reduced bone mineral density [21], and interestingly, morphometric vertebral fractures, considered the most relevant clinical manifestations of osteoporosis and bone fragility, have been reported to be highly prevalent in COVID-19 patients. In addition, it is indicated that these fractures can exert an influence on the impaired respiratory function in the medium term of patients who have recovered from the disease, which impairs their recovery and contributes to the emergence of long-COVID [17].

The molecular interactions and pathophysiological outcomes involving various human systems remain incompletely elucidated. In relation to bone health, the prognosis of infected patients may be worsened by factors such as an exacerbated cytokine storm that reduces bone mineral density, prolonged confinement associated with vitamin D deficiency, and the widespread use of corticosteroids.

Patients with OI, a rare genetic disorder of connective tissue, already present many of these risk factors, including low bone mass, altered bone remodeling, and a tendency toward skeletal fragility [5,6]. Additionally, vitamin D dysregulation is not uncommon in this population, making them potentially more susceptible to the adverse osteometabolic effects of SARS-CoV-2 infection [22,23,24]. However, despite these plausible biological connections, the impact of COVID-19 on individuals with OI remains poorly understood.

Bisphosphonates are a class of drugs widely used in the treatment of bone diseases, such as OI, by inhibiting osteoclast-mediated bone resorption. Beyond their antiresorptive action, recent evidence points to an immunomodulatory effect of bisphosphonates, with potential activity against viral antigens [25]. In a cohort of approximately seven million patients in the United States, an association was observed between bisphosphonate use and lower rates of COVID-19-related hospitalization among individuals with bone diseases compared to those not using the medication [25]. Another relevant drug for patients with bone diseases is vitamin D. Besides its fundamental role in bone metabolism, vitamin D contributes to maintaining pulmonary barrier integrity, stimulating the innate immune response, reducing the production of inflammatory cytokines, and, by blocking the nuclear factor kappa B (NF-κB) pathway, promotes an anti-inflammatory response [26].

To date, there is a notable lack of studies exploring the clinical course, severity, or outcomes of COVID-19 in individuals with rare bone disorders such as OI, especially in low- and middle-income countries. Most available reports focus on broader endocrine or metabolic conditions, without disaggregating data specific to skeletal dysplasias. Only one study, conducted in Saudi Arabia, has specifically examined patients with OI infected by SARS-CoV-2 [27]. In Brazil—a country with substantial COVID-19 burden and significant healthcare disparities—no studies have investigated how patients with OI respond to COVID-19.

Therefore, this study aims to fill this critical knowledge gap by describing the clinical and epidemiological characteristics of Brazilian patients with OI hospitalized for COVID-19 between 2020 and 2024. Understanding how this rare population fares during systemic viral infection is essential for improving clinical management, informing public health strategies, and guiding future research in the field of rare skeletal disorders.

## 2. Materials and Methods

This is a retrospective descriptive analysis study, in which the epidemiological analysis was performed based on data made available by the Open-Data-SUS (SUS, which stands for the Portuguese *Sistema Único de Saúde*) platform (https://opendatasus.saude.gov.br/, accessed on 20 March 2024), created by the Brazilian Ministry of Health to compile surveillance information on severe acute respiratory syndrome obtained by the Influenza Epidemiological Surveillance Information System (SIVEP-Gripe, which stands for the Portuguese *Sistema de Informação da Vigilância Epidemiológica da Gripe*). The database has previously been used in epidemiological studies related to COVID-19, demonstrating its significant analytical value, especially in pandemic situations that require rapid and effective governmental responses for social and economic management focused on health indicators—particularly within the public health system. However, as with any open-access administrative dataset, issues of data completeness, accuracy, and potential bias must be acknowledged.

The file was viewed using the Statistical Package for the Social Sciences (SPSS) software (IBM SPSS Statistics for Macintosh, Version 28.0). Data were extracted for the first four years of the COVID-19 pandemic, covering the period from March 2020 to March 2024, according to the following information:

(i) Demographic profile, including notification date, notification state (federation unit—States and Federal District), sex (male and female), race (White people, Black people, Asian individuals, Mixed individuals, and Indigenous peoples), place of residence (housing region: urban or rural + peri-urban), and age (years).

(ii) Data on viral infection, such as presence of hospital-acquired (nosocomial) infection, use of antivirals for the treatment of flu-like symptoms, and vaccination status against COVID-19.

(iii) Presence of comorbidities, including heart diseases, hematological disorders, Down syndrome, liver diseases, asthma, diabetes mellitus, neurological disorders, chronic respiratory disorders, immunosuppressive disorders, kidney diseases, and obesity.

(iv) Clinical signs and symptoms related to the diagnosis of severe acute respiratory syndrome, such as fever, cough, sore throat, dyspnea, respiratory discomfort, peripheral oxygen saturation <95%, diarrhea, vomiting, fatigue, and other symptoms.

(v) Need for intensive care unit (ICU) admission and need for mechanical ventilation support (invasive mechanical ventilation, noninvasive mechanical ventilation, and no need for mechanical ventilation).

(vi) The outcome criteria encompassed hospital discharge and mortality.

To ensure the accuracy of the data, the categorical variables were coded numerically, enabling the analysis of missing data, as well as descriptive statistical analysis.

The entire data set was exported from SPSS to an .xls file in order to impute the missing data using XLSTAT Statistical Software version 2024.1 for Excel. Patient characteristics with more than 40% missing data were excluded from the analysis. The NIPALS (Nonlinear Iterative Partial Least Squares) algorithm was used to impute the missing data. After imputation, XLSTAT Statistical Software generated a new data set in .xls format, which was used to conduct the statistical analyses in SPSS software, including descriptive analysis (number of individuals and percentage).

Multiple imputation was performed on the complete database, which included all patients hospitalized with COVID-19 in Brazil. After imputation, patients with OI were manually identified by two independent researchers (L.F.A.M. and F.A.L.M.). These patients were then separated from the full dataset, and their data were presented in the form of a table and a figure.

Despite these methodological steps to improve data quality, limitations persist. Data entry is performed by healthcare professionals at local institutions under heterogeneous conditions, making the dataset susceptible to underreporting, misclassification, and input errors. The Open-Data-SUS platform also lacks centralized validation mechanisms at the point of data entry. Furthermore, the database does not include certain clinically relevant information—such as genotypic data, medication use (e.g., bisphosphonates or vitamin D), and long-term outcomes—which limits the scope of the analysis.

In the present study, due to the small number of patients (N = 13) with OI, it was not possible to perform inferential analysis by comparing their characteristics with those of the remaining population. However, the discussion includes a descriptive analysis of the data, supported by findings from previous studies that used the same dataset (Open-Data-SUS).

This study was carried out according to the Declaration of Helsinki and was approved by the Institution’s Ethics Committee (Certificate of Presentation for Ethical Appreciation No. 67241323.0.0000.5514; Study approval No. 5.908.611, approval date 24 February 2023).

## 3. Results

**Figure 1:**
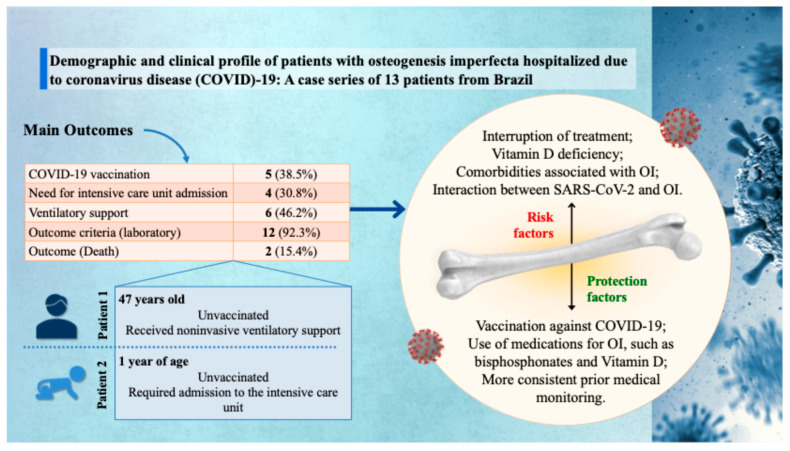
Graphical representation of the main findings from a case series of 13 patients with osteogenesis imperfecta (OI) hospitalized due to coronavirus disease (COVID)-19 in Brazil. Clinical outcomes include low vaccination coverage (38.5%), need for intensive care unit admission (30.8%), ventilatory support (46.2%), laboratory abnormalities (92.3%), and mortality (15.4%). Two illustrative cases are highlighted: an unvaccinated 47-year-old adult who received noninvasive ventilatory support, and a 1-year-old unvaccinated child who required intensive care unit admission. On the right, the infographic summarizes key risk and protective factors influencing clinical outcomes, such as treatment interruption, vitamin D deficiency, OI-related comorbidities, and the protective role of COVID-19 vaccination, specific medications (e.g., bisphosphonates and vitamin D), and consistent prior medical follow-up. SARS-CoV-2: severe acute respiratory syndrome coronavirus 2.

A total of 13 patients with OI hospitalized for SARS-CoV-2 infection were identified from the entire dataset (N = 2,078,062), with the state of Minas Gerais reporting the highest number of cases [4 (30.8%)]. Among the reported patients, 7 (53.8%) were male; 9 (69.2%) were adults and 4 (30.8%) were children. Regarding ethnicity, most self-identified as White (9; 69.2%), and all patients resided in urban areas (Table 1 and Figure 1).

Concerning disease severity, four patients (30.8%) required admission to an ICU, and six (46.2%) required noninvasive ventilatory support. Among those admitted to the ICU, only two required invasive mechanical ventilation. The clinical outcome was death in two cases (15.4%). Both patients were male, White, and had not been vaccinated against SARS-CoV-2. One was 47 years old, was not admitted to the ICU, but required noninvasive ventilation. The other was a one-year-old child who required intensive care (Table 1 and Figure 1).

## 4. Discussion

In this study, we identified 13 hospitalized patients with OI and confirmed SARS-CoV-2 infection, predominantly adults, male, and White, with all residing in urban areas. The state of Minas Gerais accounted for the highest number of cases. The most frequent clinical manifestations included fever, cough, oxygen desaturation, dyspnea, and respiratory distress. None of the patients received antiviral treatment. Comorbidities were reported in a minority of cases, including heart diseases, chronic lung diseases, and obesity. Only 38.5% had been vaccinated against SARS-CoV-2. Regarding disease severity, 30.8% required ICU admission, 46.2% required noninvasive ventilatory support, and two patients died—both unvaccinated, male, and White, including a one-year-old child and a 47-year-old adult.

It is known that SARS-CoV-2 infection leads to an exacerbated activation of the immune system, resulting in excessive production of inflammatory cytokines and intense recruitment of immune cells. This process triggers deleterious effects on the vascular barrier, capillaries, alveoli, and cell-to-cell interactions, potentially causing damage to multiple organs—including bone tissue [18,19,28]. In this context, the main observed bone impairment is the reduction in bone mineral density, mainly attributed to increased osteoclastogenesis. A study conducted by Tahtabase et al. (2021) [29], which evaluated the bone mineral density of 209 patients infected with SARS-CoV-2, demonstrated that mortality rates, ICU admission, and the need for mechanical ventilation were significantly higher among individuals with lower bone mineral density (38.1% vs. 13.0%; 33.4% vs. 21.2%, and 38.1% vs. 8.2%, respectively). The study’s multivariate analysis indicated that low bone mineral density is a strong independent predictor of mortality from COVID-19 [29].

At the beginning of the pandemic, one of the first prophylactic measures adopted to contain the spread of SARS-CoV-2 was social isolation, which led the population to remain confined in their homes. Consequently, there was a significant reduction in sun exposure, causing a drop in vitamin D levels—it is worth noting that its native form does not have biological activity. Although different studies present conflicting results regarding the impact of the pandemic on serum vitamin D levels, it is widely recognized that this micronutrient plays an essential role in modulating the immune response. Insufficient vitamin D levels have been associated with worse clinical outcomes in patients infected with SARS-CoV-2 [18,30]. In countries that adopted strict confinement measures, such as China, a difference in vitamin D levels was observed between pandemic and non-pandemic periods. In the study by Chen and Kong, for example, a significant increase in mean serum levels of 25-hydroxyvitamin D (25(OH)D) was recorded, rising from 18.14 ng/mL [IQR: 13.78–23.68] in 2022 to 19.15 ng/mL [IQR: 14.88–25.01] in 2023 [30].

Another factor that may worsen the bone prognosis of COVID-19 patients is the use of corticosteroids. Given the rapid spread of the virus and the therapeutic urgency, available medications were used based on emerging evidence. In this context, the World Health Organization (WHO) approved the use of corticosteroids as a strategy to suppress the exacerbated inflammatory response in infected patients. It has been demonstrated, for example, that the administration of dexamethasone reduced the mortality rate among patients requiring supplemental oxygen or mechanical ventilation. However, prolonged use of glucocorticoids, especially in hospitalized patients, has well-recognized adverse effects on bone metabolism. These drugs promote bone mass loss and increase fracture risk, constituting an additional vulnerability factor in individuals with preexisting osteometabolic impairment [28].

Our findings of the patients with OI hospitalized due to COVID-19 reflect important particularities compared to other populations affected by the pandemic in Brazil. First, the increased vulnerability of patients with rare diseases, such as OI, aligns with observations in other population groups with specific comorbidities. For example, in patients with Huntington’s disease, a higher risk of death was observed compared to controls without comorbidities, despite a lower prevalence of clinical signs, suggesting that these rare conditions may predispose to adverse clinical outcomes even without intense infection manifestation [31]. Furthermore, comparison with patients with Down syndrome reinforces the need for special attention to these groups, as they presented high lethality rates, greater need for intensive and ventilatory support, as well as a distinct combination of comorbidities, demonstrating that genetic and chromosomal diseases complicate the clinical picture of COVID-19 [32]. Similarly, social and racial determinants showed a strong influence on outcomes, evidenced by increased mortality among Black, Indigenous, and mixed-race populations in Brazil, highlighting the importance of social and racial context in infection prognosis [33,34]. Low representativeness and underdiagnosis in patients with rare and genetic diseases, such as those linked to the X chromosome, are also common challenges that hinder a complete assessment of COVID-19’s impact on these populations, potentially underestimating the true magnitude of the problem [35].

In a nationwide retrospective observational study conducted in England, electronic health records of 58,162,316 million people were analyzed, of which 894,396 had been diagnosed with at least one rare disease [36]. Of those, 7965 died from COVID-19, compared to the 141,287 deaths of the 58,162,316 individuals. Most individuals with rare diseases were female (55.0% vs. 50.2%), under 18 years (25.2% vs. 19.9%) or older than 70 years (28.2% vs. 13.1%). Additionally, Asian or Asian British individuals and Black or Black British individuals were reported to be the minority among other races (6.7% vs. 9.6% and 2.9% vs. 3.9%, respectively) compared to the full study population. Individuals with rare diseases were also more likely to be on the shielding list than those without rare diseases (23.5% vs. 7.0%). An analysis of the risk of COVID-19-related deaths in people with rare diseases with that in matched controls from the general population was conducted, which showed that for individuals who were vaccinated, the risk of COVID-19 related death was increased for 8 of 331 rare diseases, as for individuals who were not fully vaccinated, the risk of COVID-19 related death was increased for 28 of 331 rare diseases. Interestingly, OI was ranked second among the three rare diseases with the highest risk of COVID-19-related mortality in vaccinated individuals [36].

Regarding post-COVID-19 functional sequelae, Brazilian data indicate that disease severity is associated with greater pulmonary alterations and reduced functional capacity, reinforcing the importance of continuous clinical follow-up for at-risk patients, including those with rare diseases [37]. Additionally, underreporting of COVID-19 cases, especially among young age groups, suggests that the pandemic epidemiology in Brazil may underestimate the real impact in certain groups, complicating effective planning and targeting of public policies for vulnerable populations [38]. In chronic conditions such as cystic fibrosis, hospital mortality may vary significantly by context, with Brazilian data showing a more severe profile compared to other countries, which may reflect inequalities in access to treatment and adequate support [39].

The psychological and social impact of the pandemic must also be considered, as factors such as social isolation, behavioral changes, and resistance to preventive measures directly influenced adherence to control strategies and, consequently, clinical outcomes observed in different populations. Finally, the Indigenous population in Brazil, with its cultural and linguistic diversity, faced unique challenges, including high vulnerability to contagion, underreporting, and difficulties in accessing diagnosis and treatment, reflecting an epidemiological tragedy that requires specific emergency measures [34]. Thus, the present study contributes to knowledge about the impact of COVID-19 in populations with rare diseases, highlighting the need for targeted policies that consider the clinical and social specificities of these groups within the complex Brazilian epidemiological scenario.

When analyzing the demographic and epidemiological profile, it stands out that the Brazilian Indigenous population, estimated at approximately 897,000 individuals, presents high vulnerability due to community exposure and limited access to health care [34]. In the OI study, most of the 13 patients were adults (69.2%) and White (69.2%), residing in urban areas, unlike rural Indigenous populations. Additionally, racial groups such as Indigenous, mixed-race (*Pardos*), and Black individuals have higher COVID-19 mortality risk, similarly to the OI study, which observed higher mortality in unvaccinated patients, predominantly male, a pattern also found in other hospitalized populations [33,40].

Clinical impact and lethality show that while overall hospital lethality in Brazil was approximately 33.2%, increasing to 55.9% in patients requiring ICU and 79.3% in invasive mechanical ventilation [40], the OI study showed lower lethality of 15.4%, despite disease severity. In contrast, the Indigenous population exhibited mortality above 40% [34], and patients with neurodegenerative diseases such as Creutzfeldt-Jakob disease had even higher lethality around 83% [41], suggesting that COVID-19 worsening depends significantly on the associated comorbidity type.

Symptoms such as fever, cough, desaturation, and dyspnea were predominant both in the OI study and in Indigenous and general hospitalized populations [34,40]. Some specific symptoms, such as loss of smell, muscle pain, and sore throat, were more prevalent in COVID-19 than in other respiratory etiologies, as observed in Indigenous peoples and some OI cases [34]. Cardiovascular, pulmonary, and renal comorbidities were common and associated with worse prognosis in several studies, a situation partly observed in OI patients. Asthma, however, was described in another study as a protective factor [42], contrasting with observations in OI. Rural Indigenous pregnant women had significantly higher mortality, evidencing specific vulnerabilities not present in the OI group [34].

Regarding ventilatory support, 46.2% of OI patients required noninvasive ventilation, and 15.4% invasive ventilation, with mortality associated with this need. Similar data reinforce that invasive mechanical ventilation significantly increases death risk, a fact observed both in Indigenous populations and in the OI group [40]. Patients with neurodegenerative diseases and COVID-19 showed an even greater need for intensive support, reflecting the severity of the infection [41].

COVID-19 vaccination was identified as a protective factor in all studied groups, highlighting mortality reduction in Indigenous and general populations [40,41]. In the OI study, only 38.5% of patients were vaccinated, and both deaths occurred among the unvaccinated, reinforcing the importance of immunization to reduce pandemic impact. Social, economic, and cultural inequalities were widely highlighted as elements increasing vulnerability of Indigenous populations, with higher infection rates, mortality, and lower vaccine access [34]. The OI population, predominantly urban and White, possibly had better access to health systems, which may partially explain the lower lethality observed in this group.

Finally, the association between SARS-CoV-2 and rare neurodegenerative diseases such as Creutzfeldt-Jakob disease presents a clinical picture of rapid progression and high lethality, demonstrating that COVID-19 can significantly worsen outcomes in chronic neurological diseases, differing from the impact observed in OI patients who do not have a neurodegenerative component [41].

In Brazil, the data point to a complex scenario in which COVID-19 severity and clinical outcomes are intrinsically linked not only to pre-existing clinical conditions but also to social, racial determinants and access to healthcare services, including vaccination coverage. Therefore, public policies must consider these multiple dimensions to guarantee effective and equitable strategies in facing the pandemic, especially for vulnerable groups such as patients with rare diseases, Indigenous peoples, and carriers of complex chronic diseases.

The international literature presents only one study that investigated the clinical outcomes of patients with OI infected with SARS-CoV-2 [27]. This is a retrospective study conducted in Saudi Arabia between 2020 and 2021, which included 146 patients with a confirmed diagnosis of OI. Among them, 12 (8.2%) were diagnosed with COVID-19. Most of the infected patients (9; 75.0%) were under 18 years old, and half were male. Seven (58.3%) patients had normal bone mineral density, and none required hospitalization. Regarding the clinical presentation, 11 (91.7%) exhibited mild symptoms—such as fever, headache, myalgia, arthralgia, and cough—and a large portion were using bisphosphonates (6; 50.0%) and vitamin D supplementation (10; 83.3%). Similarly, the present study identified that Brazilian patients with OI hospitalized due to COVID-19 predominantly presented with fever and cough, and most had a favorable prognosis, being discharged from the hospital. However, unlike the study by Alshukairi et al. [27], two deaths were observed among the patients analyzed in Brazil, suggesting possible differences in clinical, epidemiological factors, or access to treatment between the populations studied.

A relevant parameter in the analysis of clinical outcomes of OI patients hospitalized for COVID-19 is the prior use of specific medications for the treatment of OI. Although the present study did not have access to information regarding the medications used by patients before or during hospitalization, it is widely recognized that bisphosphonates and vitamin D currently represent the most promising pharmacological therapies for managing the disease. In this sense, it is plausible to assume that a significant portion of OI patients regularly use these medications. This hypothesis is supported by the study of Alshukairi et al. [27], in which 92 of the 146 OI patients (63.0%) were using bisphosphonates, and 10 of the 12 patients diagnosed with COVID-19 (83.3%) were on vitamin D supplementation. Therefore, it is considered relevant to investigate in future studies whether these therapies may act as protective factors against severe progression of SARS-CoV-2 infection, especially due to their potential immunomodulatory effects and benefits to bone metabolism, which can be compromised during systemic inflammatory conditions such as COVID-19 [43].

Despite the favorable prognosis observed in most patients analyzed in this study, it is undeniable that individuals with rare diseases were particularly impacted during the COVID-19 pandemic [44]. People living with rare diseases already face numerous challenges in normal times related to diagnosis, understanding of their condition, and access to appropriate treatments. With the health emergency caused by SARS-CoV-2, this scenario became even more critical due to several factors, including: (i) disruption of regular medical consultations and follow-up; (ii) difficulties in continuing and adhering to prescribed treatments; (iii) increased psychological stress, with elevated levels of anxiety and emotional distress; and (iv) the additional risk posed by SARS-CoV-2 infection and its possible interactions with the patient’s underlying condition [45].

Regarding people with OI, the COVID-19 pandemic also had a significant impact. A study conducted between 2020 and 2021 by the Brittle Bones Society (BBS), involving 110 participants—including individuals with OI and their family members—from the United Kingdom and the Republic of Ireland, evaluated the effects of the pandemic on this population [46]. Of those interviewed, 69 (62.7%) reported postponed medical appointments, and among the 62 who detailed the impacts of this interruption, 21 (33.9%) mentioned delays in treatment and 20 (32.3%) reported increased pain. A total of 57 (51.8%) received remote care; however, only 15 (26.3%) rated these consultations as excellent. Furthermore, among the 91 participants who responded about mental health effects, 63 (69.2%) reported increased feelings of anxiety and depression during the pandemic period [46].

In our cohort, both recorded deaths occurred in unvaccinated patients, while all vaccinated individuals survived, suggesting a potential protective role of SARS-CoV-2 immunization. Although no statistical inference can be drawn due to the small sample size, this finding aligns with global evidence on the effectiveness of vaccination in reducing COVID-19 severity and mortality. It is important to note that, particularly in the early stages of the pandemic, there were substantial challenges to achieving broad vaccine coverage in Brazil, including logistical barriers, vaccine availability, prioritization protocols, and vaccine hesitancy in certain groups [47]. These factors may have disproportionately affected individuals with rare diseases, such as OI, who often face additional barriers to accessing healthcare services. We emphasize the need for public health strategies that ensure timely and equitable access to vaccination for all at-risk populations. Furthermore, future studies with larger and more diverse cohorts are essential to better assess the impact of vaccination and other prognostic determinants in individuals with rare bone diseases.

In response to the challenges faced by people with rare bone diseases, including OI, during the pandemic, the initiative “COVID-19 Helpline for Rare Bone Diseases” was created by the European Reference Network on Rare Bone Diseases (ERN BOND). Implemented in Italy, this service provided remote support with specialized knowledge on rare bone diseases, addressing general recommendations, pulmonary complications, and therapeutical guidance for both patients and healthcare professionals. The project was successful and exemplifies the importance of specialized and accessible care in public health emergencies, such as the one experienced during the COVID-19 pandemic [48].

Although the use of secondary open-access data imposes inherent methodological limitations, it also provides a unique opportunity to explore underrepresented populations—such as individuals with OI—on a national scale. To our knowledge, this is the largest, most recent, and only study available in the scientific literature to date that evaluates hospitalized patients with OI and COVID-19, adding valuable epidemiological insight. The reliance on descriptive analysis, dictated by the small sample size and the nature of the dataset, limits the possibility of causal inference. Future studies should adopt more innovative and robust research designs, including multicenter cohort studies or registries with clinical, laboratory, and genotypic data, as well as detailed records of therapeutic interventions. These approaches would allow for a more comprehensive understanding of the impact of COVID-19 on rare bone diseases and support the development of targeted healthcare strategies.

This study has several limitations. Notably, there is no information on molecular diagnostic tests or the specific pathogenic variants of the patients with OI. The data were entered by healthcare professionals from the respective institutions and are thus subject to potential recording errors and underreporting. Additionally, although the database comprises nearly two million patients, it still covers only a portion of the Brazilian population, requiring caution when extrapolating the results. The sample size of this study is small—only 13 patients—and restricted to Brazil, which limits its generalizability and prevents a continental or global perspective. As such, the findings represent only a snapshot of the clinical progression of patients with OI. The observed outcomes may also have been influenced by uncontrolled variables, such as the use of specific medications, availability of social support networks, and access to healthcare services. Furthermore, the database does not include certain clinical information relevant to this study, such as the use of bisphosphonates or vitamin D by the patients.

## 5. Conclusions

Understanding the clinical and epidemiological profile of patients with OI hospitalized due to SARS-CoV-2 infection is an important step toward developing tailored public health strategies for this vulnerable group. Although our sample was small and clinically heterogeneous, the findings offer preliminary insights into the manifestations and outcomes of COVID-19 in individuals with rare bone disorders. The observation that both recorded deaths occurred in unvaccinated patients highlights the potential relevance of vaccination in mitigating severe outcomes. However, given the limited sample size, no definitive conclusions can be drawn regarding causality, reinforcing the need for broader and more detailed investigations on the role of immunization in this population. Furthermore, the scarcity of data on the clinical course of patients with OI during COVID-19, particularly in low- and middle-income countries such as Brazil, represents a significant gap in the literature. This study underscores the importance of future research efforts to explore prognostic factors—such as vaccination status, comorbidities, and the potential role of treatments like vitamin D and bisphosphonates—in greater depth. While most patients in our analysis had favorable outcomes, the occurrence of two deaths illustrates the need for continuous clinical surveillance and individualized care protocols for patients with rare skeletal disorders, regardless of age.

## Figures and Tables

**Table 1:** Clinical and demographic characteristics of patients with osteogenesis imperfecta hospitalized due to coronavirus disease (COVID)-19 in Brazil.

Characteristics	P1	P2	P3	P4	P5	P6	P7	P8	P9	P10	P11	P12	P13
Notification date	02/23	07/21	02/21	12/22	01/21	11/21	08/20	09/20	04/21	05/21	03/22	03/22	05/23
Notification state	MG	FD	MS	MG	RS	PE	SC	PE	SP	MG	PR	MG	AL
Sex	M	F	F	F	M	M	M	F	F	F	M	M	M
Age (years)	50	58	31	22	47	60	47	<1	25	58	1	2	<1
Race	B	W	W	W	W	MI	W	MI	W	W	W	MI	W
Housing region	U	U	U	U	U	U	U	U	U	U	U	U	U
Nosocomial infection	No	No	No	No	No	No	No	No	No	No	No	No	No
Clinical signs and symptoms													
Fever	Yes	Yes	No	Yes	Yes	Yes	No	Yes	No	Yes	Yes	Yes	Yes
Cough	No	No	No	Yes	Yes	Yes	Yes	Yes	Yes	Yes	No	Yes	Yes
Sore throat	No	No	No	Yes	Yes	No	No	No	No	No	No	No	No
Dyspnea	No	No	No	No	Yes	Yes	Yes	Yes	Yes	Yes	No	Yes	Yes
Respiratory discomfort	No	Yes	No	No	Yes	Yes	Yes	Yes	No	No	No	Yes	Yes
Peripheral oxygen saturation <95%	No	Yes	No	Yes	Yes	Yes	Yes	Yes	Yes	No	No	Yes	Yes
Diarrhea	No	No	No	No	No	No	No	No	No	Yes	No	No	No
Vomiting	No	No	No	No	Yes	No	No	No	No	No	Yes	Yes	No
Fatigue	No	No	No	No	No	No	No	No	No	Yes	No	No	No
Another symptom	No	Yes	Yes	Yes	No	No	No	Yes	Yes	Yes	No	No	No
Comorbidities													
Heart diseases	No	Yes	No	No	No	No	Yes	No	No	No	No	No	No
Hematological disorders	No	No	No	No	No	No	No	No	No	No	No	No	No
Down syndrome	No	No	No	No	No	No	No	No	No	No	No	No	No
Liver diseases	No	No	No	No	No	No	No	No	No	No	No	No	No
Asthma	No	No	No	No	No	No	No	No	No	No	No	No	No
Diabetes mellitus	No	No	No	No	No	No	No	No	No	No	No	No	No
Neurological disorders	No	No	No	No	No	No	No	No	No	No	No	No	No
Chronic respiratory diseases	No	No	No	No	No	No	No	No	No	No	No	Yes	No
Immunosuppressive disorders	No	No	No	No	No	No	No	No	No	No	No	No	No
Kidney diseases	No	No	No	No	No	No	No	No	No	No	No	No	No
Obesity	No	No	No	No	Yes	No	No	No	No	No	No	No	No
Vaccinated against COVID-19	No	Yes	Yes	Yes	No	Yes	No	No	No	No	No	No	Yes
Antiviral for the treatment of flu-like symptoms	No	No	No	No	No	No	No	No	No	No	No	No	No
Required admission to the intensive care unit	No	Yes	No	No	No	No	No	No	No	No	Yes	Yes	Yes
Received ventilatory support	No	NI	No	No	No	NI	NI	No	NI	NI	No	No	NI
Outcome criteria	L	C	L	L	L	L	L	L	L	L	L	L	L
Outcome	Cure	Cure	Cure	Cure	Cure	Cure	Death	Cure	Cure	Cure	Death	Cure	Cure

AL: Alagoas; B: Black; C: Clinic; F: Female; FD: Federal District; L: Laboratory; M: Male; MG: Minas Gerais; MI: Mixed individuals; MS: Mato Grosso do Sul; NI: Noninvasive; P: Patient; PE: Pernambuco; PR: Paraná; RS: Rio Grande do Sul; SC: Santa Catarina; SP: São Paulo; U: Urban; W: White.

## Data Availability

Data are available from the authors upon reasonable request.

## Abbreviations

The following abbreviations are used in this manuscript:

%	Percentage
25(OH)D	25-hydroxyvitamin D
95% CI	95% confidence interval
ACE2	Angiotensin-converting enzyme
AL	Alagoas
B	Black
BBS	Brittle Bones Society
C	Clinic
*COL1A1*	Collagen Type I Alpha 1 Chain
*COL1A2*	Collagen Type I Alpha 2 Chain
*COVID-19*	Coronavirus disease-19
ERN BOND	European Reference Network on Rare Bone Diseases
F	Female
FD	Federal District
ICU	Intensive care unit
IQR	Interquartile range
L	Laboratory
M	Male
MG	Minas Gerais
MI	Mixed individuals
MS	Mato Grosso do Sul
NF-κB	Nuclear factor kappa B
ng/mL	Nanograms per milliliter
NI	No Invasive
OI	Osteogenesis imperfecta
P	Patients
PE	Pernambuco
*PLS3*	Plastin-3
PR	Paraná
OR	Odds ratio
SARS-CoV-2	Severe acute respiratory syndrome coronavirus 2
SIVEP-Gripe	*Sistema de Informação da Vigilância Epidemiológica da Gripe*
SPSS	Statistical Package for the Social Sciences
SUS	*Sistema Único de Saúde*
RANKL	Receptor Activator of Nuclear Factor Kappa-B Ligand
RS	Rio Grande do Sul
S	Spike
SC	Santa Catarina
SP	São Paulo
U	Urban
W	White
WHO	World Health Organization

## References

[1] Subramanian S., Anastasopoulou C., Viswanathan V.K. (2025). Osteogenesis Imperfecta. StatPearls.

[2] Warman M.L., Cormier-Daire V., Hall C., Krakow D., Lachman R., LeMerrer M., Mortier G., Mundlos S., Nishimura G., Rimoin D.L. (2011). Nosology and Classification of Genetic Skeletal Disorders: 2010 Revision. Am. J. Med. Genet. A.

[3] Aliyeva L., Ongen Y.D., Eren E., Sarisozen M.B., Alemdar A., Temel S.G., Sag S.O. (2024). Genotype and Phenotype Correlation of Patients with Osteogenesis Imperfecta. J. Mol. Diagn..

[4] Byrd J.J., White A.C., Nissen C.G., Schissel M., Van Ormer M., Velasco D., Wallace M. (2024). Genotype-Phenotype Correlations in 294 Pediatric Patients with Osteogenesis Imperfecta. JBMR Plus.

[5] Rossi V., Lee B., Marom R. (2019). Osteogenesis Imperfecta: Advancements in Genetics and Treatment. Curr. Opin. Pediatr..

[6] Van Dijk F.S., Sillence D.O. (2014). Osteogenesis Imperfecta: Clinical Diagnosis, Nomenclature and Severity Assessment. Am. J. Med. Genet. A.

[7] Botor M., Fus-Kujawa A., Uroczynska M., Stepien K.L., Galicka A., Gawron K., Sieron A.L. (2021). Osteogenesis Imperfecta: Current and Prospective Therapies. Biomolecules.

[8] Santili C., Akkari M., Waisberg G., Bastos Júnior J.O.C., Ferreira W.M. (2005). Avaliação clínica, radiográfica e laboratorial de pacientes com osteogênese imperfeita. Rev. Assoc. Med. Bras..

[9] Deguchi M., Tsuji S., Katsura D., Kasahara K., Kimura F., Murakami T. (2021). Current Overview of Osteogenesis Imperfecta. Medicina.

[10] Fernandes A.M., Rocha-Braz M.G.M., França M.M., Lerario A.M., Simões V.R.F., Zanardo E.A., Kulikowski L.D., Martin R.M., Mendonca B.B., Ferraz-de-Souza B. (2020). The Molecular Landscape of Osteogenesis Imperfecta in a Brazilian Tertiary Service Cohort. Osteoporos. Int..

[11] Holtz A.P., Souza L.T., Ribeiro E.M., Acosta A.X., Lago R.M.R.S., Simoni G., Llerena J.C., Félix T.M. (2023). Genetic Analysis of Osteogenesis Imperfecta in a Large Brazilian Cohort. Bone.

[12] Reis F.C., Alexandrino F., Steiner C.E., Norato D.Y.J., Cavalcanti D.P., Sartorato E.L. (2005). Molecular Findings in Brazilian Patients with Osteogenesis Imperfecta. J. Appl. Genet..

[13] Martins J.P., Siqueira B.A., Sansone N.M.S., Marson F.A.L. (2023). COVID-19 in Brazil: A Three-Year Update. Diagn. Microbiol. Infect. Dis..

[14] Lauwers M., Au M., Yuan S., Wen C. (2022). COVID-19 in Joint Ageing and Osteoarthritis: Current Status and Perspectives. Int. J. Mol. Sci..

[15] Puig-Domingo M., Marazuela M., Yildiz B.O., Giustina A. (2021). COVID-19 and Endocrine and Metabolic Diseases. An Updated Statement from the European Society of Endocrinology. Endocrine.

[16] Mung S.M., Jude E.B. (2021). Interplay between Endocrinology, Metabolism and COVID-19 Infection. Clin. Med..

[17] di Filippo L., Frara S., Doga M., Giustina A. (2022). The Osteo-Metabolic Phenotype of COVID-19: An Update. Endocrine.

[18] Sapra L., Saini C., Garg B., Gupta R., Verma B., Mishra P.K., Srivastava R.K. (2022). Long-Term Implications of COVID-19 on Bone Health: Pathophysiology and Therapeutics. Inflamm. Res..

[19] Harris A., Creecy A., Awosanya O.D., McCune T., Ozanne M.V., Toepp A.J., Kacena M.A., Qiao X. (2024). SARS-CoV-2 and Its Multifaceted Impact on Bone Health: Mechanisms and Clinical Evidence. Curr. Osteoporos. Rep..

[20] di Filippo L., Doga M., Frara S., Giustina A. (2022). Hypocalcemia in COVID-19: Prevalence, Clinical Significance and Therapeutic Implications. Rev. Endocr. Metab. Disord..

[21] Giustina A., Bouillon R., Binkley N., Sempos C., Adler R.A., Bollerslev J., Dawson-Hughes B., Ebeling P.R., Feldman D., Heijboer A. (2020). Controversies in Vitamin D: A Statement from the Third International Conference. JBMR Plus.

[22] Coccia F., Pietrobelli A., Zoller T., Guzzo A., Cavarzere P., Fassio A., Flodmark C.-E., Gatti D., Antoniazzi F. (2023). Vitamin D and Osteogenesis Imperfecta in Pediatrics. Pharmaceuticals.

[23] Gnoli M., Brizola E., Tremosini M., Di Cecco A., Sangiorgi L. (2023). Vitamin D and Bone Fragility in Individuals with Osteogenesis Imperfecta: A Scoping Review. Int. J. Mol. Sci..

[24] Abdrabbo M., Birch C.M., Brandt M., Cicigoi K.A., Coffey S.J., Dolan C.C., Dvorak H., Gehrke A.C., Gerzema A.E.L., Hansen A. (2021). Vitamin D and COVID-19: A Review on the Role of Vitamin D in Preventing and Reducing the Severity of COVID-19 Infection. Protein Sci..

[25] Thompson J., Wang Y., Dreischulte T., Barreiro O., Gonzalez R.J., Hanč P., Matysiak C., Neely H.R., Rottenkolber M., Haskell T. (2023). Association between Bisphosphonate Use and COVID-19 Related Outcomes. Elife.

[26] Argano C., Mallaci Bocchio R., Natoli G., Scibetta S., Lo Monaco M., Corrao S. (2023). Protective Effect of Vitamin D Supplementation on COVID-19-Related Intensive Care Hospitalization and Mortality: Definitive Evidence from Meta-Analysis and Trial Sequential Analysis. Pharmaceuticals.

[27] Alshukairi A.N., Doar H., Al-Sagheir A., Bahasan M.A., Sultan A.A., Al Hroub M.K., Itani D., Khalid I., Saeedi M.F., Bakhamis S. (2022). Outcome of COVID19 in Patients with Osteogenesis Imperfecta: A Retrospective Multicenter Study in Saudi Arabia. Front. Endocrinol..

[28] Bandeira F., Bilezikian J.P. (2022). Bone Diseases and the COVID-19 Pandemic. Arch. Endocrinol. Metab..

[29] Tahtabasi M., Kilicaslan N., Akin Y., Karaman E., Gezer M., Icen Y.K., Sahiner F. (2021). The Prognostic Value of Vertebral Bone Density on Chest CT in Hospitalized COVID-19 Patients. J. Clin. Densitom..

[30] Chen Y., Kong G. (2024). Changes in Vitamin D Status among Adults from the COVID-19 Pandemic to Post-Pandemic Normality. Front. Nutr..

[31] Martins J.P., Sciani J.M., Marson F.A.L. (2025). Huntington’s Disease in Hospitalized Patients Infected with SARS-CoV-2 in Brazil: Three-Year Update. Neurodegener. Dis..

[32] Boschiero M.N., Palamim C.V.C., Ortega M.M., Marson F.A.L. (2022). Clinical Characteristics and Comorbidities of COVID-19 in Unvaccinated Patients with Down Syndrome: First Year Report in Brazil. Hum. Genet..

[33] Sansone N.M., Boschiero M.N., Valencise F.E., Palamim C.V., Marson F.A. (2022). Characterization of Demographic Data, Clinical Signs, Comorbidities, and Outcomes According to the Race in Hospitalized Individuals with COVID-19 in Brazil: An Observational Study. J. Glob. Health.

[34] Sansone N.M.S., Mello L.S., Martins J.P., Marson F.A.L. (2025). Impact of Coronavirus Disease (COVID)-19 on the Indigenous Population of Brazil: A Systematic Review. J. Racial Ethn. Health Disparities.

[35] Boschiero M.N., Sansone N.M.S., Marson F.A.L. (2023). Hospitalized Patients with X-Linked Disease and Infected with SARS-CoV-2 in Brazil: A Serial Case Report from the First Two Years of the Pandemic. Respir. Investig..

[36] Thygesen J.H., Zhang H., Issa H., Wu J., Hama T., Phiho-Gomes A.-C., Groza T., Khalid S., Lumbers T.R., Hocaoglu M. (2025). Prevalence and Demographics of 331 Rare Diseases and Associated COVID-19-Related Mortality among 58 Million Individuals: A Nationwide Retrospective Observational Study. Lancet Digit. Health.

[37] Conti P.B.M., Ribeiro M.Â.G.O., Gomez C.C.S., Souza A.P., Borgli D.S.P., Sakano E., Pascoa M.A., Severino S.D., Castilho T., Marson F.A.L. (2025). Pulmonary and Functional Hallmarks after SARS-CoV-2 Infection across Three WHO Severity Level-Groups: An Observational Study. Front. Med..

[38] Palamim C.V.C., Siqueira B.A., Boschiero M.N., Marson F.A.L. (2023). Increase in COVID-19 Underreporting among 3,282,337 Brazilian Hospitalized Patients Due to SARS: A 3-Year Report and a Major Concern for Health Authorities. Travel. Med. Infect. Dis..

[39] Marques L.S., Boschiero M.N., Sansone N.M.S., Brienze L.R., Marson F.A.L. (2023). Epidemiological Profile of Hospitalized Patients with Cystic Fibrosis in Brazil Due to Severe Acute Respiratory Infection during the COVID-19 Pandemic and a Systematic Review of Worldwide COVID-19 in Those with Cystic Fibrosis. Healthcare.

[40] Palamim C.V.C., Camargo T.M., Valencise F.E., Marson F.A.L. (2025). Evaluation of the Case Fatality Rate in 2 031 309 Hospitalised Brazilian Patients Due to COVID-19: An Observational Study of the First 3 Years of the Pandemic in Brazil. BMJ Public Health.

[41] Silva A.E.F., Costa P.T., Mello L.S., Marques L.F.A., Dos Santos V.S., Marson F.A.L. (2025). A Serial Case Report of Hospitalized Patients with Creutzfeldt-Jakob Disease Due to Coronavirus Disease (COVID)-19 in Brazil: A Four-Year Profile. J. Neurol. Sci..

[42] Sansone N.M.S., Valencise F.E., Bredariol R.F., Peixoto A.O., Marson F.A.L. (2022). Profile of Coronavirus Disease Enlightened Asthma as a Protective Factor against Death: An Epidemiology Study from Brazil during the Pandemic. Front. Med..

[43] Shah K., Varna V.P., Sharma U., Mavalankar D. (2022). Does Vitamin D Supplementation Reduce COVID-19 Severity?: A Systematic Review. QJM.

[44] Nguengang Wakap S., Lambert D.M., Olry A., Rodwell C., Gueydan C., Lanneau V., Murphy D., Le Cam Y., Rath A. (2020). Estimating Cumulative Point Prevalence of Rare Diseases: Analysis of the Orphanet Database. Eur. J. Hum. Genet..

[45] Zybarth D., Brandt M., Mundlos C., Inhestern L. (2023). Impact of the COVID-19 Pandemic on Health Care and Daily Life of Patients with Rare Diseases from the Perspective of Patient Organizations—A Qualitative Interview Study. Orphanet J. Rare Dis..

[46] Smyth D., Hytiris M., Kelday C., McDonnell C., Burren C., Gardner A., Mills L., Parekh S., Semler O., Stewart A. (2022). Patient-Reported Experience of Clinical Care of Osteogenesis Imperfecta (OI) during the COVID-19 Pandemic. Front. Public Health.

[47] Boschiero M.N., Palamim C.V.C., Marson F.A.L. (2021). The Hindrances to Perform the COVID-19 Vaccination in Brazil. Hum. Vaccin. Immunother..

[48] Brizola E., Adami G., Baroncelli G.I., Bedeschi M.F., Berardi P., Boero S., Brandi M.L., Casareto L., Castagnola E., Fraschini P. (2020). Providing High-Quality Care Remotely to Patients with Rare Bone Diseases during COVID-19 Pandemic. Orphanet J. Rare Dis..

